# RNA-Seq reveals that overexpression of TcUBP1 switches the gene expression pattern toward that of the infective form of *Trypanosoma cruzi*

**DOI:** 10.1016/j.jbc.2023.104623

**Published:** 2023-03-17

**Authors:** Karina B. Sabalette, José R. Sotelo-Silveira, Pablo Smircich, Javier G. De Gaudenzi

**Affiliations:** 1 Instituto de Investigaciones Biotecnológicas, Universidad Nacional de San Martín - Consejo Nacional de Investigaciones Científicas y Técnicas, General San Martín, Prov. de Buenos Aires, Argentina; 2 Escuela de Bio y Nanotecnologías (EByN), Universidad Nacional de San Martín, General San Martín, Prov. de Buenos Aires, Argentina; 3 Department of Genomics, Instituto de Investigaciones Biológicas Clemente Estable, Montevideo, Uruguay; 4 Instituto de Biología, School of Sciences, Universidad de la República, Montevideo, Uruguay

**Keywords:** trypanosome, RNA–protein interaction, RNA-binding protein, gene regulation, RNA regulon

## Abstract

Trypanosomes regulate gene expression mainly by using posttranscriptional mechanisms. Key factors responsible for carrying out this regulation are RNA-binding proteins, affecting subcellular localization, translation, and/or transcript stability. *Trypanosoma cruzi* U-rich RNA-binding protein 1 (TcUBP1) is a small protein that modulates the expression of several surface glycoproteins of the trypomastigote infective stage of the parasite. Its mRNA targets are known, but the impact of its overexpression at the transcriptome level in the insect-dwelling epimastigote cells has not yet been investigated. Thus, in the present study, by using a tetracycline-inducible system, we generated a population of TcUBP1-overexpressing parasites and analyzed its effect by RNA-Seq methodology. This allowed us to identify 793 up- and 371 downregulated genes with respect to the wildtype control sample. Among the upregulated genes, it was possible to identify members coding for the *TcS* superfamily, *MASP*, *MUCI*/*II*, and *protein kinases*, whereas among the downregulated transcripts, we found mainly genes coding for ribosomal, mitochondrial, and synthetic pathway proteins. RNA-Seq comparison with two previously published datasets revealed that the expression profile of this TcUBP1-overexpressing replicative epimastigote form resembles the transition to the infective metacyclic trypomastigote stage. We identified novel *cis*-regulatory elements in the 3′-untranslated region of the affected transcripts and confirmed that UBP1m, a signature TcUBP1 binding element previously characterized in our laboratory, is enriched in the list of stabilized genes. We can conclude that the overall effect of TcUBP1 overexpression on the epimastigote transcriptome is mainly the stabilization of mRNAs coding for proteins that are important for parasite infection.

Trypanosomes are interesting models to study unusual mechanisms of gene expression regulation. Unlike most eukaryotes, trypanosomatids lack control at the level of transcription initiation for each individual gene. In contrast, transcription by RNA polymerase II is polycistronic and transcript synthesis initiates from a few sites on each chromosome (1, 2, 3). Individual mature mRNAs are generated by 5′ trans-splicing (4) and 3′ polyadenylation (5). Owing to these biological constraints, these microorganisms control protein levels mainly by posttranscriptional events. The fate of mRNAs in the cell depends on the set of RNA-binding proteins (RBPs) associated with them, and these molecular interactions can also be organized into larger mRNP complexes forming stress granules or P-bodies (6). Among the critical aspects of mRNA metabolism are 5′ and 3′-end processing (7), nuclear export (8), mRNA stability (9), and translation (10, 11, 12). Over the years we have contributed, in part, to a better understanding of these mechanisms in *Trypanosoma cruzi*, an early branching eukaryotic unicellular parasite causing Chagas disease (8, 13, 14, 15, 16, 17, 18, 19). Particularly, the first RNA-Seq transcriptome and translatome for this parasite showed that translation regulation plays a critical role in governing gene expression profiles during *T. cruzi* differentiation (10). We and other authors have reported some of the molecular mechanisms that might operate to explain this regulation (8, 16, 20). At all these regulatory points, RBPs can intervene as crucial *trans*-acting factors and mediate parasite differentiation in both *T. cruzi* and *Trypanosoma brucei* (21, 22).

The present study focused on *T. cruzi* U-rich RBP 1 (TcUBP1), one of the first trypanosome RNA-recognition motif (RRM)-containing proteins described. TcUBP1 is an exclusive trypanosomal RBP having a single RRM (23) with the characteristic β_1_α_1_β_2_β_3_α_2_β_4_ fold. It is expressed in all stages of the parasite life cycle and regulates the abundance of a large number of genes containing U-rich elements (19, 24). Some of the ribonucleoprotein complexes containing TcUBP1 are developmentally regulated, as determined by profile expression of target transcripts and RT-PCR analysis of coimmunoprecipitated RNAs (17, 18).

The ability of *T. cruzi* to survive in the mammalian host is in part due to the expression of a plethora of surface proteins and signaling genes, which include the trans-sialidase and trans-sialidase like (TcS) superfamily, mucins, and mucin-associated surface proteins, among others (25, 26). In previous studies on partners of the TcUBP1–mRNP complex by *in vivo* RBP immunoprecipitation, we found several transcripts encoding *TcS* proteins (24). Interestingly, TcUBP1, in synchrony with nutritional deficiency, is known to mediate differentiation of *T. cruzi* epimastigotes into infective metacyclic trypomastigotes (27), by coordinating a timely developmental program (28). *TcS* members are surface glycoprotein-coding genes expressed only in trypomastigote forms, but the *in vivo* interaction of TcUBP1-*TcS* RNAs occurs in both replicative and infective cells. In this regard, ectopic overexpression of TcUBP1 in replicative forms resulted in >10-fold upregulated expression of numerous *TcS* mRNAs and changes in their subcellular localization from the posterior zone to the perinuclear region of the cytoplasm, as is typically observed in the infective stages. This fact has led to the hypothesis that TcUBP1 can promote a switch toward profile expression of infective trypomastigotes in *T. cruzi* by increasing the mRNA levels and translation rates of an RNA regulon for trypomastigote surface glycoproteins during parasite development. The posttranscriptional paradigm of RNA regulons was first posited by Keene and Lager almost 2 decades ago (29, 30, 31) and suggests that, by recognizing structural and/or sequence RNA elements, cells can coregulate subsets of transcripts with a shared physiological function.

For an RBP of interest, identifying the *in vivo* binding sites is a critical step toward understanding its function. However, the complete influence of TcUBP1 overexpression in the determination of the parasite transcriptome is not known and its precise binding sites have not been described. Thus, the aim of the present study was to perform an RNA-Seq analysis on epimastigote samples overexpressing UBP1.

## Results

### Identification of differentially expressed genes after TcUBP1 overexpression

**Figure 1:**
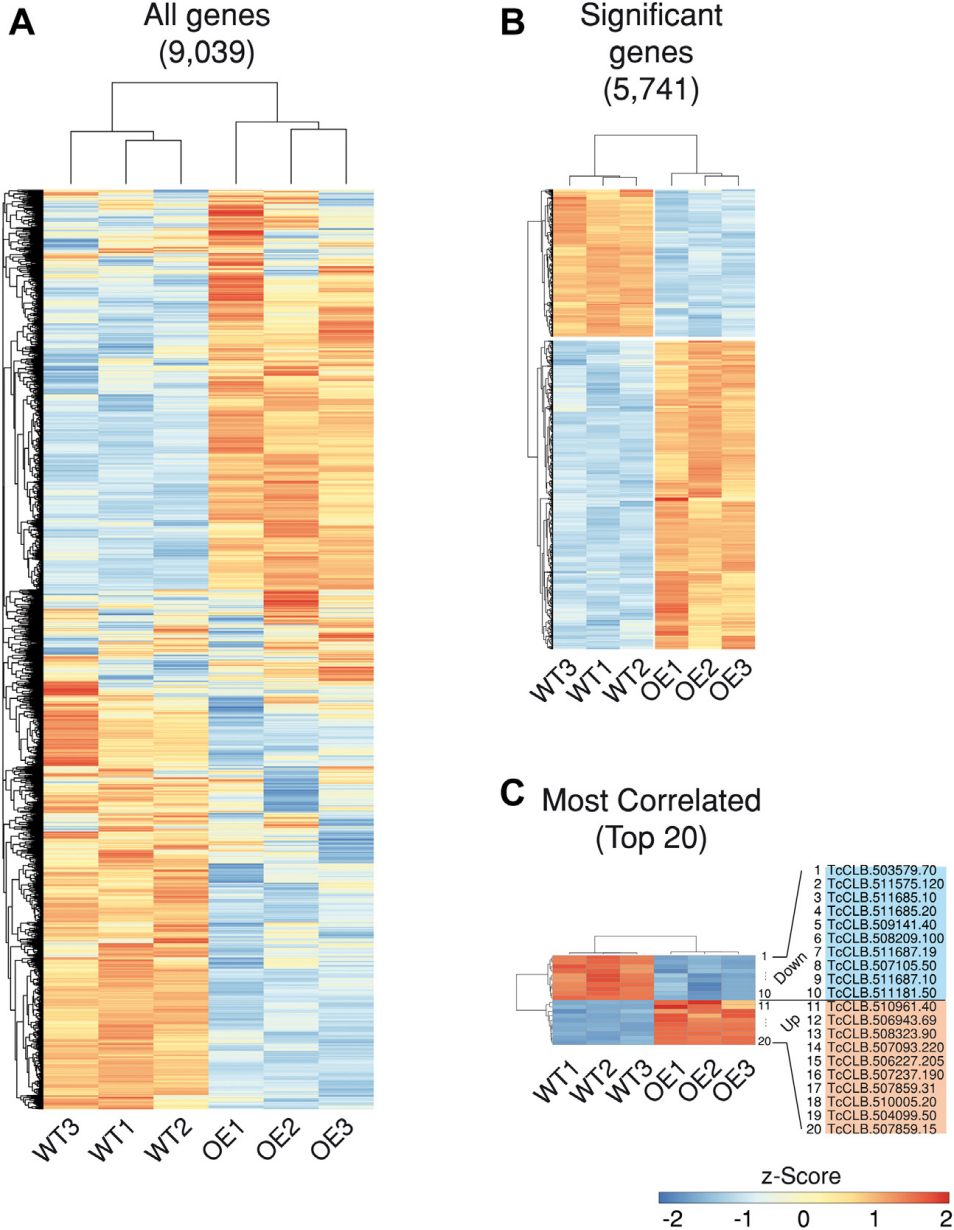
**Hierarchical clustering of differentially expressed genes in UBP1-OE samples compared with wildtype epimastigotes defined by DESeq2 with FDR less than 0.05.** Groups on vertical represent the clustered genes based on gene expression, the horizontal line represents the single gene and color of the line indicates the gene expression in UBP1-OE. *A*, heatmap and complete linkage clustering using all replicates per group, 5741 genes were clustered. *B*, heatmap of 1164 significant genes with |log2 fold change|>1 with 793 upregulated and 371 downregulated genes. *C*, most correlated samples (n = 20 genes). The Z-score scale bar represents relative expression ± SD from the mean.

To gain comprehensive insights into the regulatory role of TcUBP1, we analyzed the impact of TcUBP1 overexpression on the *T. cruzi* CL-Brener transcriptome. For this, TcUBP1-GFP-induced epimastigotes (UBP1-OE) or control wildtype samples (WT) were subjected to RNA-Seq analysis (see Experimental procedures). After assembly and annotation, we identified a total of 9039 genes (Fig. 1*A*). The expression levels of each gene of the UBP1-OE and WT populations were calculated by mapping clean read sets onto the reference transcriptome of the CL Brener Esmeraldo-like strain (TriTrypDB-59_TcruziCLBrenerEsmeraldo-like_Genome.fasta). The data from different libraries were normalized using the normalization method in the software package DESeq2 (32).

The distribution pattern of transcript expression in UBP1-induced *versus* WT parasite populations was analyzed in detail. Results showed that 64% of the total genes (5,741) were significantly expressed, with false discovery rate (FDR)-adjusted *p* values lower than 0.05, and that 13% of the genes (1,164) were differentially expressed (|log2 fold change|>1, FDR-adjusted *p* value < 0.05; File S1). The percentages of up- and downregulated genes in UBP1 tetracycline-induced parasites were 8.8% and 4.1%, respectively. In addition, the expression patterns for all genes (A), for the significantly expressed genes (B), and for the most correlated genes (*i.e.*, genes that were found overexpressed in one sample and underexpressed in the other and vice versa) (C), in control and OE parasites are shown in Figure 1. The light blue, white, and orange colors indicate less expressed, medium-level expressed, and highly expressed genes, respectively (Fig. 1*A*). By analyzing the complete expression profile of the up- and downregulated genes, we concluded that the global effect of UBP1-OE is mostly to stabilize the transcriptome, since nearly ∼800 genes were 2-fold upregulated and less than half of the genes were 2-fold downregulated (|log2 fold change| > 1, FDR-adjusted *p*-value < 0.05) (Fig. 1*B*).

**Figure 2:**
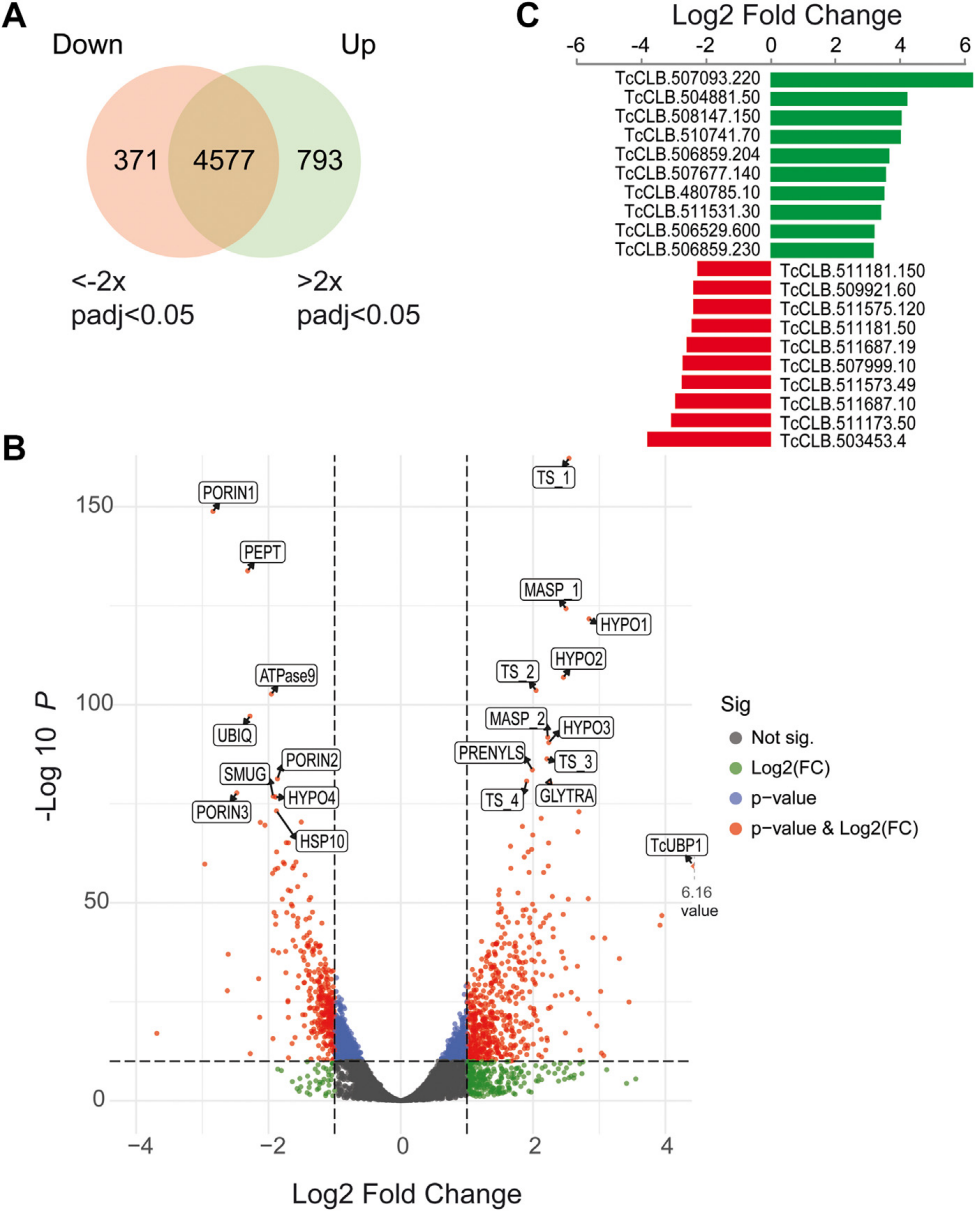
**Venn diagram and volcano plot of differentially expressed genes.***A*, Venn diagram representing the total number of differentially expressed genes between UBP1-OE and WT. UBP1 upregulated (log2 >1, FDR <0.05) or downregulated (log2 <1, FDR <0.05) genes are shown. *B*, volcano plot showing the differential expression analysis of genes in UBP1-OE and wildtype parasites. Black and red dots show nonsignificant and significant differentially expressed genes, respectively. TcUBP1 and most significant up- and downregulated genes are labeled. UBP1, the most expressed gene, is plotted out of scale with and x-value of 6.16 log2 fold change. Upregulated: TS_1 (TcCLB.504099.50); MASP_1 (TcCLB.507237.190); HYPO1 (TcCLB.507859.31); HYPO2 (TcCLB.507859.15); TS_2 (TcCLB.510005.20); MASP_2 (TcCLB.506965.130); HYPO3 (TcCLB.506227.205); PRENYLS (TcCLB.507879.10); TS_3 (TcCLB.506211.150); TS_4 (TcCLB.511311.20); GLYTRA (TcCLB.510071.30); downregulated: PORIN1 (TcCLB.511687.10); PEPT (TcCLB.511181.50); ATPase9 (TcCLB.503579.70); UBIQ (TcCLB.511575.120); PORIN2 (TcCLB.504225.20); PORIN3 (TcCLB.511687.19); SMUG (TcCLB.511685.10); HYPO4 (TcCLB.503897.120); HSP10 (TcCLB.508209.100). *C*, chart of top 10 most up/downregulated genes based on log2 fold change.

A Venn diagram was generated to show a representation of the differentially expressed genes mentioned above (Fig. 2*A*). This included 793 upregulated and 371 downregulated genes in TcUBP1-induced samples (File S1). The number of upregulated genes in UBP1-OE parasites was two times higher than that of downregulated genes. In addition, results showed that 33 genes, most of which coded for hypothetical proteins, were expressed exclusively in UBP1-OE samples, and that 11 genes, mostly related to the chromosome organization process, were expressed exclusively in WT parasites (File S2). As expected, TcUBP1 (TcCLB.507093.220) was 71 times higher in the OE samples (FDR-adjusted *p* value = 1.1E-58), showing the largest difference between the OE and WT samples. This value reflected the expected overexpression of TcUBP1 as a consequence of the pTcINDEX induction with tetracycline. A volcano plot of gene expression in UBP1-induced and WT parasites is shown in Figure 2*B*, where significantly expressed genes are separated from the nonsignificantly expressed genes by different color codes. The 20 most statistically significant up- and downregulated genes are toward the top, labeled with gene symbols together with TcUBP1. Also, the Top 10 list with the most differentially over- or underexpressed genes (based on fold change values) is shown in Table 1 and also depicted in Figure 2*C*.

**Table 1:** Top 10 of most up- and downregulated genes after UBP1 overexpression based on fold change values

Regulation	Product name	GeneID	Fold change
Upregulated genes:			
Up	RNA-binding protein UBP1, putative	TcCLB.507093.220	71.51
	ABC transporter, putative	TcCLB.504881.50	17.88
	Mucin-associated surface protein (MASP), subgroup S008	TcCLB.508147.150	15.45
	STE/STE11 serine/threonine-protein kinase, putative	TcCLB.510741.70	15.14
	Hypothetical protein, conserved	TcCLB.506859.204	11.79
	NLI interacting factor-like phosphatase, putative	TcCLB.507677.140	10.93
	Serine/threonine kinase, putative	TcCLB.480785.10	10.63
	Hypothetical protein, conserved	TcCLB.511531.30	9.85
	Metacyclin II, putative	TcCLB.506529.600	8.63
	Hypothetical protein, conserved	TcCLB.506859.230	8.46
Downregulated genes:			
Down	Hypothetical protein, conserved	TcCLB.511181.150	−4.44
	Dispersed gene family protein 1 (DGF-1), putative	TcCLB.509921.60	−4.86
	JAB1/Mov34/MPN/PAD-1 ubiquitin protease, putative	TcCLB.511575.120	−4.86
	Mitochondrial processing peptidase, beta subunit, putative	TcCLB.511181.50	−4.96
	Mitochondrial outer membrane protein porin, putative (fragment)	TcCLB.511687.19	−5.58
	Dispersed gene family protein 1 (DGF-1), putative	TcCLB.507999.10	−6.11
	Hypothetical protein	TcCLB.511573.49	−6.15
	Mitochondrial outer membrane protein porin, putative	TcCLB.511687.10	−7.41
	Mucin-associated surface protein (MASP), subgroup S081	TcCLB.511173.50	−7.78
	Protein of unknown function (DUF1242), putative	TcCLB.503453.4	−12.91

### TcUBP1 overexpression leads to upregulation of cell-surface trypomastigote glycoproteins and downregulation of ribosomal and mitochondrial proteins

**Figure 3:**
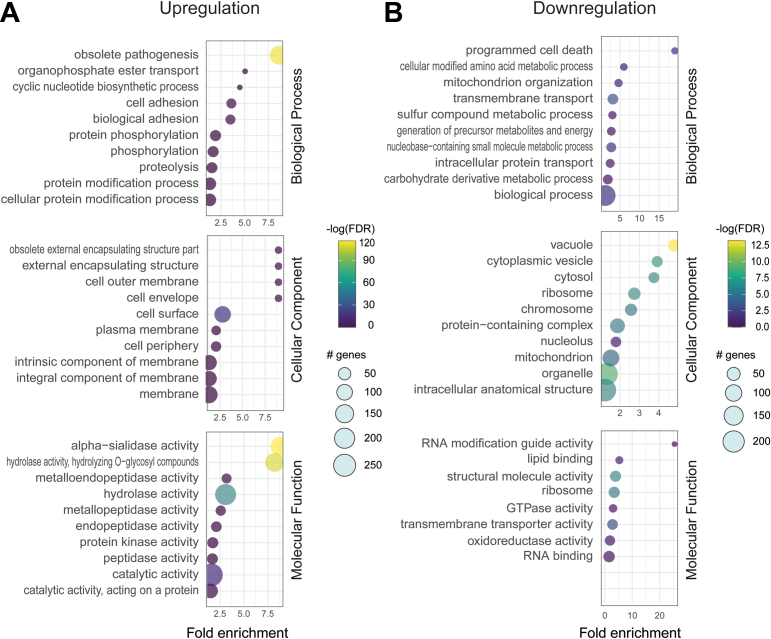
**Differentially expressed genes enrichment analysis.** Gene Ontology (GO) classification of differentially expressed genes, the graph shows up to 10 GO terms with most genes annotated. First, second, and third charts indicated GO terms clustered in the biological process, cellular component and molecular function terms, respectively. The size of the dots diameter indicates the number of differential genes; color depth indicates significance; abscissa indicates enrichment abundance; and the ordinate indicates different pathways. *A*, upregulation. *B*, downregulation.

Gene ontology (GO) analyses using TriTrypDB performed on genes over- and underexpressed in UBP1-OE parasites showed a distribution of 18 and 107 GO overrepresented terms, respectively. The enrichment chart was plotted showing each significant GO term and the percentage of genes present in our differentially expressed genes compared with the background for each category (Fig. 3). The complete distribution is provided in File S3. A plot for all the three GO domains, biological process, molecular function, and cellular process, is presented in Figure 3*A* (upregulation) and Figure 3*B* (downregulation). The GO analysis of differentially expressed genes with significant differences revealed that they are involved in critical biological processes and cellular components, such as pathogenesis, cell adhesion, and protein phosphorylation (in the case of upregulated genes), and in ribosomes, GTPase activity, and mitochondria (in the case of downregulated genes).

DAVID (Database for Annotation, Visualization, and Integrated Discovery) enrichment analysis classified all the enriched protein domains into three categories: InterPro, Pfam, and Smart. DAVID annotation products were recovered using the online GeneID Conversion tool. Of 793 genes in the upregulated group, 791 were accepted by DAVID for the analysis and assigned to 9 clusters, whereas all the 371 genes in the downregulated group were accepted and assigned to 8 clusters (File S4). Based on FDR-adjusted *p* values, among the top enriched domains for the upregulated group, the trypanosome sialidase, protein kinase, and RNA-binding domains had the largest number of genes (Table 2). For the downregulated genes, the most abundant classes were found to be the mitochondrial substrate/solute carrier, 40S ribosomal protein, and small GTP-binding protein domains. The results obtained using the graphical tool of the ShinyGO web application are shown in Fig. S1.

**Table 2:** Gene ontology clusters defined by DAVID server

Upregulated genes (OE > WT)					Downregulated genes (OE < WT)				
ID	Database	Domain term	Gene count	*p*_Value	ID	Database	Domain term	Gene count	*p*_Value
IPR008377	INTERPRO	Trypanosome sialidase^a^	149	3.3E-77	IPR018108	INTERPRO	Mitochondrial substrate/solute carrier^a^	6	4.2E-3
IPR000719	INTERPRO	Protein kinase, catalytic domain^a^	29	3.3E-3	IPR027500	INTERPRO	40S ribosomal protein S1/3, eukaryotes^a^	3	3.2E-3
PF00076	PFAM	RNA recognition motif. (a.k.a. RRM, RBD, or RNP domain)	9	1.2E-1	IPR005225	INTERPRO	Small GTP-binding protein domain^a^	6	3.7E-2
IPR006186	INTERPRO	Serine/threonine-specific protein phosphatase…	5	9.6E-2	IPR021053	INTERPRO	Dispersed gene family protein 1, C terminus	8	1.7E-1

List of top four clusters enriched in the up- or downregulated genes after UBP1 overexpression.

a Enrichment score > 1.5, *p* value <0.031.

**Figure 4:**
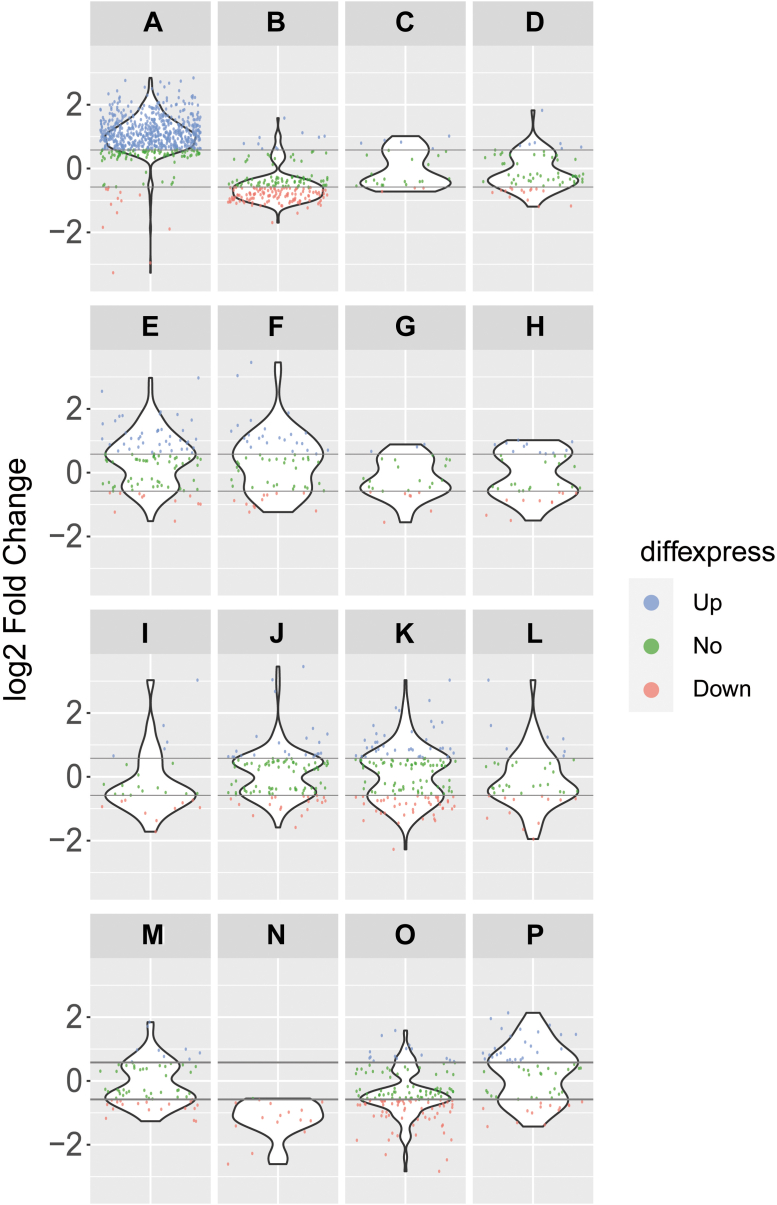
**Violin plots displaying the expression distribution of the genes within 16 different functional categories in the UBP1-OE cell transcriptome.** Two transversal *gray lines* separate three groups of expression intensity: low expressed genes in *red* (<-1.5-fold UBP1-OE/WT [<-0.58 log2 fold change]), highly expressed genes in *blue* (>1.5× UBP1-OE/WT [>0.58 log2 fold change]), and middle expressed *genes in green* (>-1.5-fold and <1.5-fold UBP1-OE/WT [>-0.58 and <0.58 log2 fold change]). Categories in the figure are *A*) membrane glycoproteins, *B*) ribosome proteins, *C*) RNA transcription, *D*) cell division and DNA synthesis, *E*) protein kinases, *F*) protein phosphatases, *G*) flagellar proteins, *H*) chaperones, *I*) lipid, fatty acid, and ATP biosynthesis, *J*) biogenesis, cell organization, and cellular motors, *K*) cellular signaling and processing, *L*) ATPase, *M*) glycolysis and carbohydrates metabolism, *N*) N-DGF, *O*) mitochondrial transcripts, and *P*) RNA-binding proteins.

We then investigated transcript expression by carrying out a comparative analysis of several functional gene groups. Based on the data presented above, we manually classified the majority of sequences obtained from UBP1-OE parasites into 16 general categories: cell-surface glycoproteins (A), ribosomal proteins (B), RNA transcription (C), cell division and DNA synthesis (D), protein kinases (E), protein phosphatases (F), flagellar proteins (G), chaperones (H), lipid, fatty acid and ATP biosynthesis (I), biogenesis, cell organization and cellular motors (J), cellular signaling and processing (K), ATPases (L), glycolysis and carbohydrate metabolism (M), disperse gene family proteins (N), mitochondrial transcripts (O), and RNA-binding proteins (P). The overall gene distribution of transcripts among these groups was analyzed using violin plots showing expression values (log2 fold change OE/WT) (Fig. 4).

Results confirmed that among the most abundant transcripts in the UBP1-OE transcriptome are those coding for cell-surface glycoproteins, protein kinases/phosphatases, and RNA-binding proteins (Fig. 4, *A*, *E*, *F* and *P*) and that among the least abundant transcripts are those coding for ribosomal proteins, mitochondrial transcripts, and some Dispersed Gene Family hits (Fig. 4, *B*, *N* and *O*), with the cluster of ribosomal proteins having the highest number of downregulated hits. The dispersed gene family is large, with many of its members predicted to have transmembrane domains and reported to be more abundant in the amastigote stage than in trypomastigotes and epimastigotes (33).

**Figure 5:**
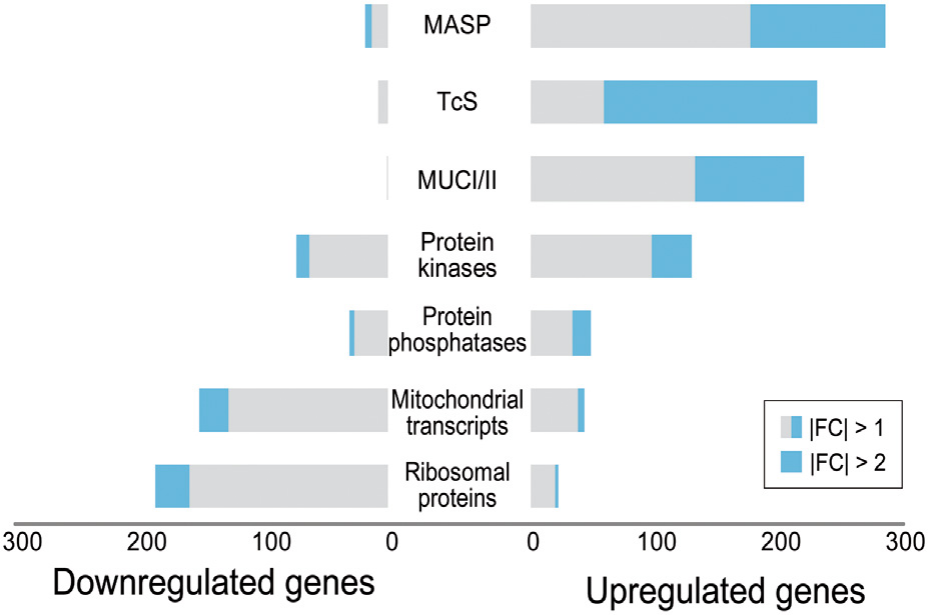
**Bars chart displaying the number of up- and downregulated genes within seven categories in the UBP1-OE transcriptome.** Categories in the figure are *MASP*, *mucin-associated surface proteins*; *TcS, trans-sialidase/trans-sialidase like*; *MUCI/II, mucin genes; Protein kinases, Protein phosphatases, Mitochondrial transcripts,* and *Ribosomal proteins*.

Clearly, the most abundant cluster among the upregulated genes in UBP1-OE samples was that of surface membrane-associated proteins. Within this group, we identified 171 *trans-sialidase/trans-sialidase-like* genes, 108 *mucin-associated surface proteins*, and 88 *mucins* (Fig. 5). Particularly, 6 of these transcripts, TcCLB.504099.50 (TS_1), TcCLB.507237.190 (MASP_1), TcCLB.510005.20 (TS_2), TcCLB.506965.130 (MASP_2), TcCLB.507879.10 (TS_3), and TcCLB.511311.20 (TS_4), were among the 10 most significantly upregulated RNAs (labeled in Fig. 2*B*; *p* value < 1E-80 and log2 fold change > 1.9). Notably, we also observed upregulation of three *trans-sialidase-like* mRNAs that had been previously reported to be upregulated in UBP1-OE parasites (28): TcCLB.506455.30 (GP85 [*trans-sialidase*, group II, putative], log2 fold change = 2.36, FDR-adjusted *p* value = 5.43e-16), TcCLB.510163.60 (C71 [*trans-sialidase*, group V, putative], log2 fold change = 3.16, FDR-adjusted *p* value = 3.71e-57), and TcCLB.508285.60 (SA85 [*trans-sialidase*, group II, putative], log2 fold change 2.61, FDR-adjusted *p* value = 9.88e-33) (Fig. S2). These three transcripts harbor a known structural TcUBP1 RNA-binding element in their 3′-UTRs, previously described in our laboratory and termed UBP1m (24). In addition, in the upregulated list, we observed 33 protein kinases and 15 protein phosphatases (see Discussion).

By contrast, many mRNAs coding for ribosomal and mitochondrial proteins were downregulated in UBP1-OE parasites. We observed transcriptional downregulation of 27 *ribosomal protein*-*coding* genes and 24 mitochondrial transcripts (Fig. 5). Particularly, 5 of these genes were among the 10 significantly downregulated transcripts (*p* value < 1E-75 and log2 fold change < −1.9): PORIN1 (TcCLB.511687.10), PEPT (TcCLB.511181.50), ATPase9 (TcCLB.503579.70), PORIN2 (TcCLB.504225.20), and PORIN3 (TcCLB.511687.19). In this Top 10 list, we also found the known TcUBP1-mRNA target *TcSMUGS* (TcCLB.511685.10) (Fig. 2*B*), as has already been reported (28, 34).

### The expression profile of UBP1-OE epimastigotes resembles that of the transcriptome of trypomastigote infective stages

We then performed a comparative transcriptomic analysis using the RNA-Seq data obtained from Smircich *et al.* (35) and Li *et al.* (36) to compare the expression profiles of TcUBP1-overexpressing parasites with those of the four *T. cruzi* stages. In order to compare between sets of RNA-Seq data from different experiments, we used an ad hoc pipeline to map the reads from these laboratories with the reference Esmeraldo-like CL Brener genome and then compared the fold change of the expression values. We calculated the percentage of regulated transcripts in UBP1-OE parasites among the most up- and downregulated genes in a pairwise comparison between the metacyclic trypomastigote (MT), cell-derived trypomastigote (Trypo), epimastigotes (Epi), and amastigotes (Ama) stages. We clustered the different fold change values for each pairwise comparison into groups of up- and downregulated genes with >1.5-, >2-, >2.83-, >4-, or >8-fold change differences between two stages (UBP1-OE *versus* Epi, MT *versus* Epi, Trypo *versus* Epi, Trypo *versus* Ama, and Epi *versus* Ama).

**Figure 6:**
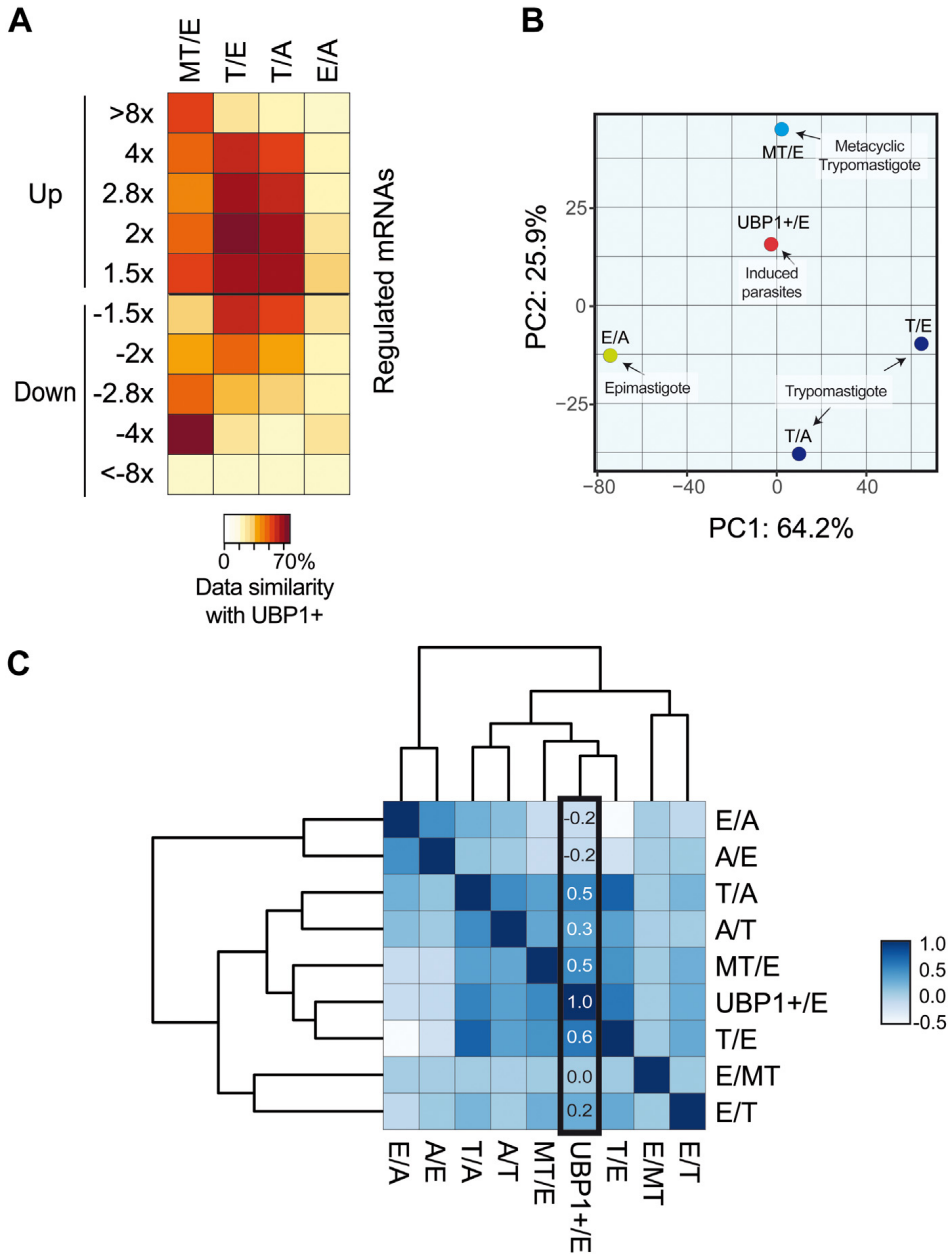
**Comparison of UBP1-OE transcriptome with RNA-Seq datasets of infective forms.***A*, heatmap representation of the percentages of shared genes between UBP1-OE and different pairwise comparisons. Data of genes with >1.5-, 2-, 2.8-, 4-, or 8-fold change differences were extracted from Smircich *et al.* (10) for column MT/E (metacyclic trypomastigote *versus* epimastigote) and Li *et al.* (36) for columns T/E (trypomastigote *versus* epimastigote), T/A (trypomastigote *versus* amastigote), and E/A (epimastigote *versus* amastigote). The *brown*/*orange* color indicates a high correlation, whereas the *yellow color* indicates a low correlation. *B*, principal component (PC) analysis plot displaying the same samples as in *A*, along PC1 and PC2, which describe 64% and 25.9% of the variability, respectively. PC analysis was applied to 1737 genes with log2 fold change data for all the pairwise comparisons. *C*, hierarchical clustering was performed using R based on Poisson distance. A/E (amastigote *versus* epimastigote), A/T (amastigote *versus* trypomastigote), E/MT (epimastigote *versus* metacyclic trypomastigote), E/T (epimastigote *versus* trypomastigote). The *dark blue color* indicates a high correlation, whereas the *light blue/white color* indicates a low correlation.

When analyzing the whole range (<4- to >8-fold change), we found that the UBP1-OE transcriptome showed highest similarity with the Trypo/Epi and MT/Epi datasets (genes overrepresented in Trypo or MT with respect to Epi). The expression profile of UBP1-OE coincided 43.0% with the MT/Epi and 43.9% with the Trypo/Epi ratios. Particularly, for the upregulated genes (>1.5- to >4-fold change), the Trypo/Epi comparison showed >60% similarity to UBP1-OE. The third dataset that was more similar to UBP1-OE was Trypo/Ama, which also displayed average percentage values of 58% in the upregulated genes. No significant coverage was found for any of the up- or downregulated transcripts in the Epi/Ama comparison. The similarity between datasets indicates that the transcriptome of UBP1-induced parasites has an expression profile that resembles that of the trypomastigote and metacyclic trypomastigote infective forms (ANOVA with post hoc Tukey test, *p* value = 0.00509). This can be visualized by different statistically significant colored clusters in the heatmap depicted in Figure 6*A* (Tukey multiple comparisons: MT/Epi - Epi/Ama, *p* value = 0.0121; Trypo/Ama - Epi/Ama, *p* value = 0.0096; and Trypo/Epi - Epi/Ama, *p* value = 0.0417).

Next, we obtained fold change values for 1737 genes from the RNA-Seq experiments (File S5). This RNA-Seq expression table was used to perform a principal component analysis (PCA) to compare the dispersion of the different datasets. The horizontal axis (PC1) describes 64.2% of the variability, and, considering this component, the sample UBP1-OE/Epi is distinctly located closer to the MT and Trypo experiments than to Epi/Ama. Thus, similar to that shown in Figure 6*A*, this analysis showed that the expression profile of the UBP1-OE population is more similar to that of the infective stages (MT/Epi, Trypo/Epi, and Trypo/Ama) than to that of the replicative stage (Epi/Ama) (Fig. 6*B*).

These expression values were then used to calculate the Pearson correlation of all the samples, to which we also added the expression values of Ama/Epi, Ama/Trypo, Epi/Trypo, and Epi/MT. The column corresponding to UBP1-OE/Epi is boxed. Again, the highest correlation was observed with the Trypo/Epi (0.5576), Trypo/Ama (0.4972), and MT/Epi (0.4610) datasets (Fig. 6*C*). No significant correlation was found between UBP1 and any of the remaining RNA-Seq datasets. The analysis of shared genes, PCA, and correlation between the different experiments analyzed showed that UBP1-overexpressing parasites have an expression profile that resembles that of infective forms of *T. cruzi*.

### Identification of *cis*-elements in the 3′-UTR of genes regulated by TcUBP1 overexpression

We next searched this transcriptome for the occurrence of a known structural UBP1 RNA-binding element, UBP1m, previously described in our laboratory (24), and also *de novo* sequence motifs. The most abundant mRNA targets previously identified for TcUBP1 encode for energy metabolism and cell-surface membrane glycoproteins. As mentioned above, the transcriptome analysis showed that, in UBP1-OE cells, these groups are either over- or underrepresented: cell-surface trypomastigote glycoproteins are upregulated and mitochondrial transcripts coding for proteins related to energy metabolism are downregulated.

With this result in mind, we decided to analyze how many of the mRNAs impacted by TcUBP1 overexpression could be direct interacting targets. For this purpose, we used the presence of the characteristic binding element UBP1m (24) as a target criterion. We then evaluated the motif coverage of UBP1m in the up- and downregulated genes in the UBP1-OE sample. We looked at all transcripts that were expressed with fold changes ranging from < -8X to > 8×.

**Figure 7:**
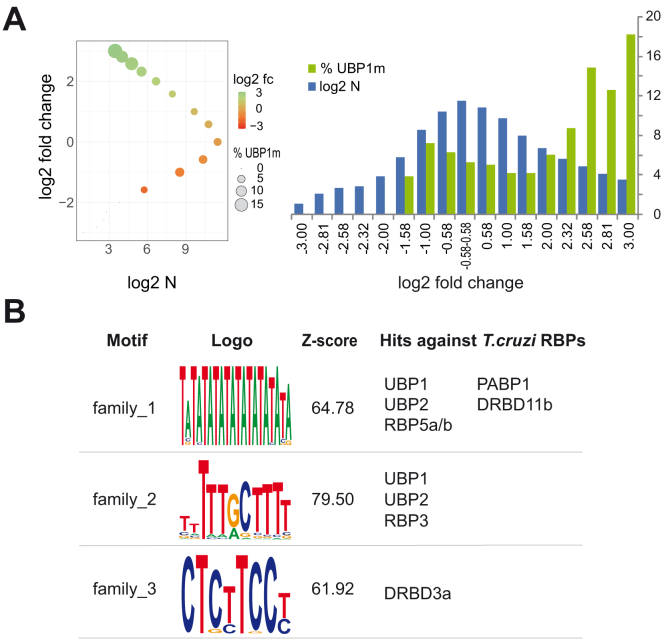
**RNA motifs identified in transcripts upregulated by TcUBP1 overexpression.***A*, *bubble* (*left*) and bar (*right*) charts of the percentage of sequences harboring the experimental motif UBP1m within the 3′-UTRs in each group (from <-3 to >3-log2 fold change OE *versus* WT). *B*, predicted RNA elements, logo consensus graphic, and Z-score in the group of upregulated genes (>4-fold change) defined by TRAWLER. Hits predicted to bind the motifs were obtained by a homemade bioinformatic pipeline^1^ (see text).

The downregulated genes showed no significant differences in the presence of the UBP1m motif. Similarly, we observed that not regulated genes, found to be between the −1.5-fold and 1.5-fold categories (log2 fold change = −0.58 to log2 fold change = 0.58), presented a 5% UBP1m coverage (147 out of 2829). However, as the fold change increased in the upregulated genes, the abundance of the UBP1m element also increased (see Fig. 7*A*). Genes with > 5-fold upregulation (log2 fold change > 2.32) showed an 8% (4 of 46) presence of UBP1m, genes upregulated >6-fold showed 15% motif coverage (4 of 27), and genes with >8-fold showed the highest motif coverage (18%; 2 of 11). This is consistent with the idea that the UBP1m motif might have a stabilizing effect on the mRNAs containing it. This all makes sense given that the UBP1m was originally detected in UBP1-immunoprecipitated mRNAs. Therefore, mRNAs stabilized by UBP1 (and containing the motif) were easily purified, whereas those destabilized and possibly containing other elements did not precipitate. We concluded that the UBP1 binding motif was enriched in the group of upregulated genes (with log2 fold change values >2.32) compared with all the remaining groups (ANOVA analysis, post hoc Tukey, *p* value = 1.47E-05).

After that, by using a cutoff value of 4-fold change, we detected 89 up- and 14 downregulated genes in UBP1-OE parasites. For each group, a length of 350 nt downstream from the coding sequences was downloaded using TcruziDB to obtain sequences resembling the 3′-UTR, in agreement with data previously reported for trypanosomes (37). The upregulated list was partitioned into two subgroups: set 1 (composed of 45 genes) and set 2 (composed of 44 genes). Thus, we first searched motifs in set 1 and then in set 2. To this end, we ran the motif prediction tool TRAWLER (http://trawler.monash.edu.ar) with default parameters, using set 1 as input sequences and the downregulated group as the background list. Results showed three candidate motifs: family_1 (5′-TVTMTATATATATATATABR-3′, Z-score: 64.78), family_2 (5′-NNTTTRCTTTB-3′, Z-score: 79.50) and family_3 (5′-CTCYTSCY-3′, Z-score 61.92). The WebLogo representation indicated that the family_1 motif is rich in AT content, whereas the family_3 motif has a CT-rich sequence composition (Fig. 7*B*). When the relative frequencies of these motifs within the 3′-UTR of both the input (set 1) and the control (set 2) datasets were examined using the FIMO software, an enrichment of all elements was observed: family_1 showed 7.47 total hits/kbps in set 1 and 5.24 total hits/kbps in set 2; family_2 showed 3.86 total hits/kbps in set 1 and 3.11 total hits/kbps in set 2; and family_3 showed 1.64 total hits/kbps in set 1 and 1.03 total hits/kbps in set 2.

We next generated a homemade bioinformatic pipeline to predict the RNA binding of motifs to sequence proteins based on integrated published data sources.^1^ For this purpose, we used the sequence elements as a query to search for proteins with the ability to bind them according to Tomtom motif analysis (https://meme-suite.org/meme/tools/tomtom, MEME Suite). Thus, heterologous interacting proteins were predicted as binders by searching against the RNAcompete database composed of 244 eukaryotic RBPs, with a *p* value < 0.004 (38, 39). Then, highly similar *T. cruzi* proteins were identified as binders of these motifs by using the previous RNAcompete hits as queries in BLASTP searches against a *T. cruzi* RBP database composed of 285 sequences with RRM (23), zinc finger, PUF, Alba, KH, and PIWI domains (22) (blastp E_value < 1E-08 and subject coverage ≥ 50%, File S6). After this step, an RNA–protein interaction prediction software that uses only primary sequence information was systematically run on all previously obtained candidates to select those reliable interactions that have binding probabilities > 0.5, by using an SVM classifier (40). The results are shown in Figure 7*B*, with five putative RBP targets for family_1 (UBP1, UBP2, RBP5A/B, PABP1, and DRBD11B), three for family_2 (UBP1, UBP2, and RBP3), and only one for familiy_3 (DRBD3A). Of note, TcUBP1 was predicted to bind both family_1 and family_2 RNA motifs. Not surprisingly, the polypyrimidine tract–binding protein DRBD3A/PTB1 (TcCLB.506649.80) was predicted to bind the C/T-rich family_3 sequence. Moreover, we validated our predictions by using molecular docking experiments. To this, we used the TcUBP1 protein structure predicted by the AlphaFold database (Q4E1N5.pdb) and obtained the 3D structures of the RNA motifs with the 3DRNA software (https://bio.tools/3dRNA). We then ran the HDOCK docking server (41) and checked the RNA–protein interactions using the transcript sequences UAUAUAUAUAUAUAUAUAUA (as family_1 RNA ligand) and UUUGCUUUU (as family_2 RNA ligand). As positive controls, we used two experimental sequences reported to be binders of TcUBP1: UBP1m (5′-UGGCGCAUCCAUGCCUGGAUGCGCCG-3′) (24) and UBP1m28 (5′-UUUUGGAGGAAGUUUUUUUUGGGG-3').^2^ In all the cases, we obtained HDOCK confidence scores >0.75, suggesting that these molecular interactions occur (Table 3). Taken together, these results suggest that these two family_1 and family_2 motifs, identified in the 3′-UTRs of the upregulated transcripts, might be involved in the interaction with TcUBP1.

**Table 3:** Protein–RNA docking of TcUBP1 and *cis*-regulatory elements

RNA motif	Nucleotide sequence (from 5′ to 3′)	Confidence score
family_1	UAUAUAUAUAUAUAUAUAUA	Model_1 =0.9366, very high probability
family_2	UUUGCUUUU	Model_1 =0.8745, very high probability
UBP1m	UGGCGCAUCCAUGCCUGGAUGCGCCG	Model_1 =0.9421, very high probability
UBP1m28	UUUUGGAGGAAGUUUUUUUUGGGG	Model_1 =0.90, very high probability

RNA motifs, nucleotide sequence, and HDOCK confidence score obtained for model 1 using TcUBP1 AlphaFold predicted structure Q41NE5 as receptor and RNA element (family_1, family_2, UBP1m or UBP1m28) as ligand.

## Discussion

Trypanosomes harbor epigenetic modifications that change between their life cycle stages (42). Nonetheless, it is broadly accepted that transcription by RNA polymerase II in these pathogens deviates from the standard eukaryotic paradigm. In *T. cruzi*, there is no dedicated promoter for each gene, resulting in polycistronic transcription, and thus gene expression regulation depends heavily on large posttranscriptional networks (43). In the present work, we used an *in vitro* system based on the inducible expression of a GFP-tagged UBP1 to monitor transcriptome changes during the differentiation of *T. cruzi* from noninfectious epimastigotes to infectious metacyclic trypomastigotes. In addition, we performed the bioinformatic analysis of two RNA-Seq samples, with three biological replicates each (Fig. S3), highlighting the differential transcript abundance and providing a data source to understand how this parasite becomes infectious.

Several lines of evidence support the role of certain RBPs as key regulators of trypanosome differentiation (21, 22, 44, 45, 46, 47, 48, 49, 50, 51, 52). In a previous work, we showed that TcUBP1 binds to structural binding elements highly enriched in transcripts coding for surface cell virulence factors associated with the metacyclic trypomastigote developmental stage (24). In epimastigotes, translation of these transcripts is diminished and thus localized in the posterior zone of the cell until a stimulus such as the ectopic expression of TcUBP1-GFP triggers the metacyclogenesis program, upregulating and mobilizing these trypomastigote stage-specific mRNAs to polysomes (28).

In the present study, when analyzing exclusive transcripts in a given experimental condition, we found that the mRNAs expressed only in WT parasites are mostly related to chromosome organization, while those exclusively expressed in TcUBP1-GFP parasites code for mitochondrial RNA processing (File S2). Our results also evidenced that 3035 of 9039 genes (34%) showed significant differences in the mRNA steady-state levels in TcUBP1-OE parasites compared with WT parasites (|log2 fold change| > 0.58, FDR 0.05). It can be easily noticed that a high number of genes coding for trypomastigote cell-surface glycoproteins are stabilized in the transcriptome of the UBP1-transgenic epimastigotes (Fig. 5). The transcriptome difference between UBP1-OE *versus* Epi-WT is similar to the one observed between the quiescent infective metacyclic trypomastigote MT *versus* Epi (Fig. 6).

In agreement with the data described for the closely related parasite *T. brucei* (53), in the present study, we obtained a profile expression resembling that of the quiescent infectious trypomastigote parasites by overexpressing a single RRM protein, UBP1, in noninfectious epimastigotes. This conclusion is based upon two results. On the one hand, we focused on the upregulation of RNA abundances of numerous cell-surface trypomastigote glycoproteins, including members of the *TcS* superfamily (Figs. 4*A* and 5). This UBP1-OE transcriptome confirms our data on the glycoprotein RNA regulon of TcUBP1-expressing parasites (28). On the other hand, we noticed that the genes coding for ribosomal proteins were downregulated in the UBP1-OE parasites (Figs. 4*B* and 5). This decrease in the number of ribosomal protein-coding mRNAs is consistent with the translational repression previously reported for metacyclic trypomastigotes (10, 54). Thus, distinctive gene expression hallmarks of the trypomastigote stage (55) are detected in the transcriptome of UBP1-overexpressing epimastigotes. These results further support posttranscriptional control as a critical regulatory mechanism required for parasite differentiation.

It has been reported that translation is strongly regulated during the *T. cruzi* cell cycle, causing variation in specific protein levels (56). In humans, multifunctional RBPs can regulate more than a single aspect of RNA metabolism. Schneider-Lunitz and coworkers have identified dozens of RBPs that influence mRNA abundance and translation efficiency of their targets (57). TcUBP1 could be also acting with this dual functionality. Previous results of our laboratory have demonstrated that an endogenous TcUBP1 fraction is associated with polysomes (58), and other researchers have also found TcUBP1 by means of a polysome proteomics approach (59). Regarding this, and as a consequence of TcUBP1 overexpression in epimastigotes, we also observed a change in the subcellular localization of cell-surface trypomastigote glycoprotein-coding transcripts, resembling the typical distribution of the metacyclic trypomastigote infective stage (28). Moreover, in our previous work, we also detected that trypomastigotes derived from TcUBP1 transgenic epimastigotes have an increased capacity for infection, an effect that has already been seen to be associated with increased protein expression of surface glycoproteins (55, 60). All these observations could also suggest a possible regulatory role of TcUBP1 in the translation rate of trypomastigote-specific mRNAs. To investigate the degree of protein synthesis regulation, future proteomics and ribosome profiling researches should be performed.

According to our present results, after UBP1 overexpression, more protein kinases than protein phosphatases are affected. In *T. brucei*, the MAP kinase MAPKL1 (Tb927.10.10870) regulates proteins involved in mRNA metabolism (61), whereas, in UBP1-OE parasites, six CMGC family protein kinases with sequence similarities to MAPKL1 are upregulated. Thus, the transcripts of these protein kinases could be part of the downstream cascade involved in the phosphorylation network of *T. cruzi*. In contrast, the transcript levels of the TcAMPKs involved in autophagy and parasite nutrient sensing (62) do not seem to be regulated by TcUBP1.

Gene regulatory networks provide key strategies to identify RNA regulons and candidate RBPs for functional studies and/or molecular targets for disease control (63, 64, 65, 66). The short sequence elements identified in this work could be signature marks for clusters of differentially upregulated genes (Fig. 7). In a preliminary computational work, we described a community of an RNA–protein interaction network composed of 26 *T. cruzi* RRM proteins (23) and 5 potential 3′ UTR regulatory motifs (Table 4).^1^ Notably, among these proteins, we found UBP1, UBP2, RBP5A/B, and TcPABP1, which are five of the seven different *trans*-factors identified for family_1 and family_2 *cis*-regulatory sequences. Regarding the mRNA expression levels of RBPs in TcUBP1-GFP-expressing parasites, we observed that 15 genes were 3-fold upregulated and that 5 genes were 2-fold downregulated (see File S7). Among the upregulated RBP genes, two were associated with TcUBP1 by being part of the same RNA–protein community described above: TcRBP9A (TcCLB.511127.10) and TcRBP26A (TcCLB.506795.10). These two proteins may be controlled by TcUBP1 and could provide positive feedback by coregulating, together with TcUBP1, mRNA targets related to the trypomastigote-specific form. TcUBP1 is expressed in all the life cycle stages of *T. cruzi* and is involved in the formation of distinct regulatory complexes. TcUBP1 has been previously reported as an interacting partner of the cytoplasmic DRBD2–mRNP complex in epimastigotes, together with UBP2, DRBD3, and PABP2, among others (67). This mRNP complex has a different RBP composition, and possibly a different function, than the previous RNA–protein network mentioned above.

**Table 4:** Community of RNA–protein interactions

Gene symbol	Type	Gene ID/Sequence	Reference^a^
TcUBP1	RRM protein	TcCLB.507093.220	(34)
TcUBP2	RRM protein	TcCLB.507093.229	(73)
TcRBP5	RRM protein	A) TcCLB.511481.55 B) TcCLB.504005.6	(17)
TcRBP7	RRM protein	A) TcCLB.506565.4 B) TcCLB.506565.8 C) TcCLB.508145.30 D) TcCLB.508145.20 E) TcCLB.508145.10 F) TcCLB.504243.10	(74)^a^
TcRBP9	RRM protein	A) TcCLB.511127.10 B) TcCLB.511481.70	(75)
TcRBP23B	RRM protein	TcCLB.507711.40	(76)
TcRBP26	RRM protein	A) TcCLB.506795.10 B) TcCLB.509937.60	(76)
TcRBP37	RRM protein	A) TcCLB.504085.30 B) TcCLB.507089.70	(76)
RBP40	RRM protein	TcCLB.506565.12	(77)
TcDRBD5B	RRM protein	TcCLB.507025.50	(76)
TcDRBD7	RRM protein	A) TcCLB.507873.30 B) TcCLB.510689.60	(76)
TcMRD1	RRM protein	A) TcCLB.503897.90 B) TcCLB.509561.110	(76)
TcPABP1	RRM protein	TcCLB.506885.70	(78)
-	RNA-binding protein, putative	TcCLB.511837.129	(22)
-	RNA-binding protein, putative	TcCLB.511837.138	(22)
RBP3m12	RBP3 binding element	5-AAGCGAAAGUGCAGAGAAUUGCUUUUUGUUU-3	(24)
UBP1m26	UBP1 binding motif	5-GCAGGAaAGUCGCGUUGUUUUUUUGG-3	(24)
UBP1m28	UBP1 binding motif	5-UUUUGGAGGAAGUUUUUUUUGGGG-3	(24)
m04144	Endocytosis binding element	5-auGCuUGUUAUUGuUUaCucAUGaCGaUGAGaGCaU-3	(17, 18)
m04130	SNARE int. in vesicular transport binding element	5-CugucugccUgugUcugUGcgcaggcgggcaG-3	(17, 18)

a For RBP7, the reference corresponds to the heterologous protein from the African trypanosome, *Trypanosoma brucei*.

By overexpressing TbRBP6 in noninfectious procyclic trypanosomes, Kolev and co-workers recapitulated *in vitro* the generation of infective metacyclic forms observed in the *tsetse* fly (21). Similarly, forced expression of TbRBP10 in procyclic forms induces differentiation to bloodstream forms (46). Since the *T. cruzi* homologues of these two regulators are among the RBP upregulated genes listed in File S7, TcRBP6A (TcCLB.506693.30) and TcRBP10 B (TcCLB.510507.50), it is tempting to speculate that TcUBP1 is upstream in the regulatory cascade that triggers parasite differentiation.

In summary, the transcriptome data presented here obtained by overexpressing TcUBP1-GFP in the noninfectious epimastigote *T. cruzi* stage provide a comprehensive picture of the mRNA steady-state level of the differentiation process toward the infective stage. Our results deepen the knowledge of previous reports of our laboratory and show that the levels of TcUBP1 trigger a posttranscriptional regulatory program that occurs during parasite differentiation, to transform replicative epimastigotes into infective quiescent metacyclic trypomastigotes.

## Experimental procedures

### Plasmid construction, parasite cultures, and transfection

The DNA construct pTcINDEX-TcUBP1-GFP previously used in Sabalette *et al*. (28) was used for parasite transfections. Protein expression values in Tet+ induced epimastigote samples after 96 h were determined relative to noninduced controls (Tet-) by Western blot analysis of GFP levels normalized to total protein loading, as measured by Coomassie Blue staining. *T. cruzi* epimastigotes, from the CL Brener strain, were cultured in BHT medium containing 10% heat-inactivated fetal calf serum (BHT 10%) at 28 °C. All parasite cultures were performed in plastic flasks without shaking, unless otherwise stated. Parasites were transfected by electroporation subsequently with pLew vector and pTcINDEX constructions and selected with 500 μg/ml of G418 and 250 μg/ml Hygromycin. For induction of recombinant proteins from the pTcINDEX vector, parasites were incubated in BHT 10% containing 0.5 μg/ml tetracycline for 96 h at 28 °C with shaking.

### RNA preparation and RNA-Seq

Total RNA was prepared from approximately 10^7^ epimastigote uninduced cells and 4-day Tet+ induced cells that express TcUBP1. RNA from three biological replicates was prepared using the TRIzol reagent from Invitrogen according to the manufacturer’s instructions. The quality of RNA samples was checked on 1% agarose gel and quantified using NanoDrop 2000 spectrophotometer (Thermo Scientific). Additional quality assessment for the integrity of RNA samples, isolation of poly (A)+ mRNA, library preparation, and sequencing on DNBSeq platform were performed at the BGI Americas Corporation.

### Overall quality parameters of the RNA-Seq data

The RNA-Seq bioanalyzer library profile of both samples was generated on Agilent 2100 instrument. The samples were next used for paired-end (PE) deep sequencing and the libraries were sequenced using 2 × 100 PE chemistry on DNBSeq platform for generating ∼5.3 GB of data per sample. After trimming of low-quality sequences, in total, ∼24M reads (∼11 GB) were obtained for each UBP1-OE and WT samples. To minimize genetic heterogeneity we choose the reference genome CL Brener Esmeraldo-like strain (TriTrypDB-59_TcruziCLBrenerEsmeraldo-like_Genome.fasta), which has a genome size of 32.53 Mbp. The obtained mapped read numbers for UBP1-OE and WT samples were 42,880,869 and 42,539,799, respectively.

### Read processing and data analysis

Read processing and data analysis were performed. The short reads less than 50 bases were dropped to exterminate the sequencing artifacts, and the quality of reads was evaluated using FASTQC toolkit (score >35) (68). The high-quality reads were *de novo* assembled using bowtie2 with parameter --very sensitive-local. Samtools were used to index the output, and the quantitative assessment of reads was performed with featureCounts with parameters '-p -t "CDS" -g "ID" -T 40' (69). PCA was performed to ensure the quality of data (Fig. S3). Differential gene analysis was conducted using DESeq2 (32). The obtained count value was used to identify the differentially expressed gene transcripts using the criteria of at least 2-fold change (|log2 fold change| >1) in the sequence count between OE and WT samples and the Benjamini–Hochberg FDR adjusted *p* value < 0.05. The Fragments Per Kilobase of transcript per Million mapped reads (FPKM) values for each transcript were log-transformed and normalized, which was subsequently used to calculate the matrix distance with Euclidean distance and complete-linkage methods. The R statistics package pheatmap was used to construct the heatmap (https://cran.r-project.org/web/packages/pheatmap.html). The differentially expressed genes were used for GO terms/KEGG pathway enrichment analyses using hypergeometric test equivalent to one-tailed Fisher’s exact test with a FDR value of 0.05 using TriTrypDB. Volcano, GO enrichment, and violin plots were constructed using R with the package ggplot2 (69, 70).

### Functional annotation of gene lists

GO analysis was carried out for the differentially expressed genes from the TriTrypDB database (http://www.tritrypdb.org). The GO sequence distribution was analyzed for all the three GO domains: biological processes, molecular function, and cellular component. All the genes for *T. cruzi* were taken as reference set and the differentially expressed genes for both lists were taken as test set (up- or downregulated after UBP1-OE). The GO annotations were extracted and visualized as bubble charts using ggplot2 in R (69, 70). Also, to categorize gene lists into overrepresented functionally related groups, DAVID (Database for Annotation, Visualization and Integrated Discovery, version 6.8) functional annotation clustering tool was used (71). Groups with an “enrichment score” (ES) > 1.5 (defined as the minus logarithm of the geometric median of *p* values) were considered significant (72).

## Data availability

RNA-Seq raw data files used in this study are available as FASTQ files of 100-bp paired-end reads in the National Center for Biotechnology Information (NCBI) Sequence Read Archive (SRA) database with the following study number: PRJNA907231.

## Supporting information

This article contains supporting information: Files S1-S7 and Figures S1-S3.

## Conflict of interest

The authors declare that they have no conflicts of interest with the contents of this article.

## Footnotes

1 J. G. De Gaudenzi, unpublished results.

2 J. G. De Gaudenzi and A. C. C. Frasch, unpublished results.
