# Supplementary material for: RNA-Seq reveals that overexpression of TcUBP1 switches the gene expression pattern toward that of the infective form of Trypanosoma cruzi
Source: J Biol Chem. 2023 Mar 17;299(5):104623. doi: 10.1016/j.jbc.2023.104623 (PMC10141520; doi:10.1016/j.jbc.2023.104623)
Supplement: Supplemental data [file mmc1.pdf]

RNA-Seq reveals that overexpression of TcUBP1 switches the gene expression pattern towards that of the infective form of *Trypanosoma cruzi*

Karina B. Sabalette[1], José R. Sotelo-Silveira[2,3], Pablo Smircich[2,3], Javier G. De Gaudenzi[*1]

From the [1] Instituto de Investigaciones Biotecnológicas, Universidad Nacional de San Martín-Consejo Nacional de Investigaciones Científicas y Técnicas, General San Martín, 1650, Prov. de Buenos Aires, Argentina; [2] Department of Genomics, Instituto de Investigaciones Biológicas Clemente Estable, Av. Italia 3318, Montevideo, CP 11600, Uruguay; [3] Instituto de Biología, School of Sciences, Universidad de la República, Montevideo, Uruguay.

Running title: *Transcriptome of T. cruzi UBP1-overexpressing parasites*

*To whom correspondence may be addressed: Javier G. De Gaudenzi; jdegaudenzi@iib.unsam.edu.ar

Keywords: trypanosome, RNA-protein interaction, RNA-binding protein, gene regulation, RNA regulon.

SUPPORTING INFORMATION

Material included:

- Figure S1
- Figure S2
- Figure S3
- Supplemental File 1
- Supplemental File 2
- Supplemental File 3
- Supplemental File 4
- Supplemental File 5
- Supplemental File 6
- Supplemental File 7

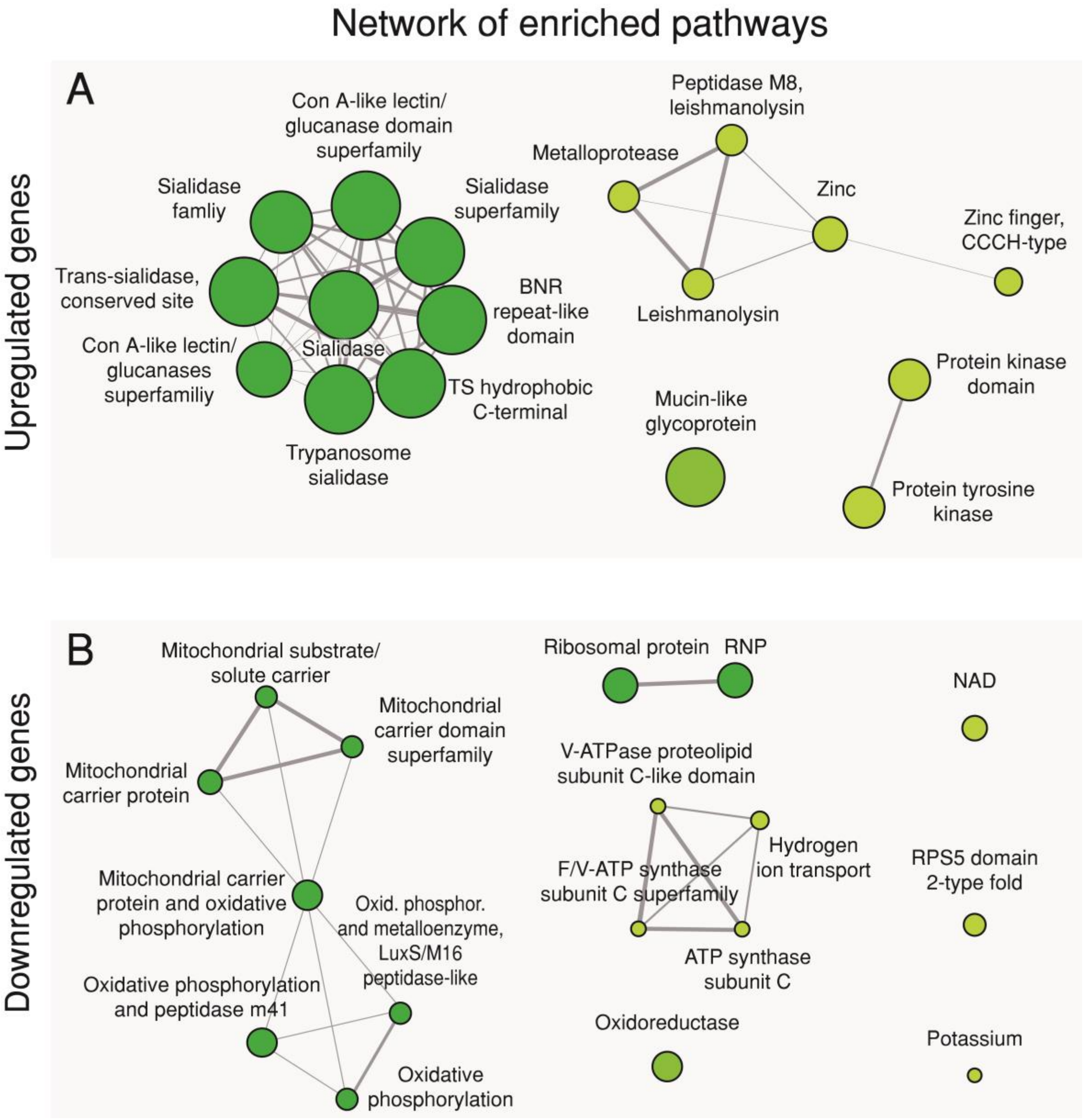

Figure S1. Significantly enriched biological process of differentially expressed genes in UBP1-OE parasites defined by ShinyGO. A) Enriched biological process of up-regulated genes. B) Enriched biological process of down-regulated genes. Gene numbers are represented by the size of the circles in each pathway.

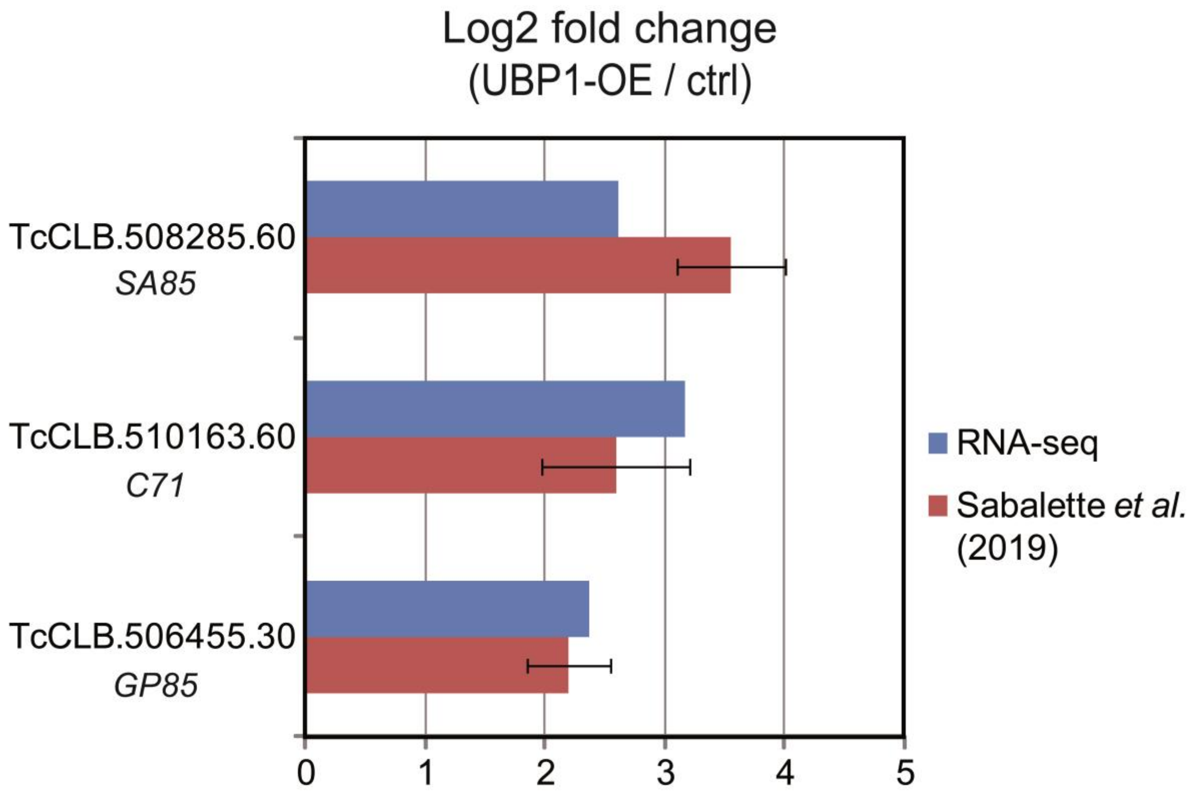

Figure S2. Up-regulation of other *trans-sialidase-like* genes previously identified after UBP1 overexpression.

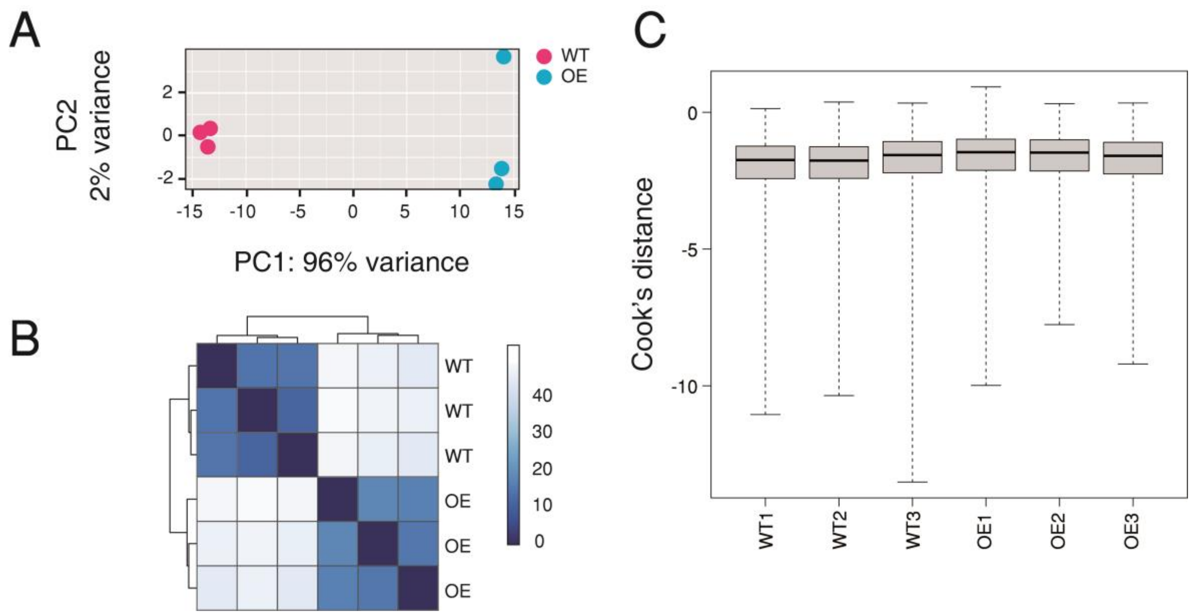

Figure S3. Assessing inter- and intragroup variability. (A) PCA plot displaying all 6 samples along PC1 and PC2, which describe 96% and 2% of the variability, respectively, within the expression data set. PC analysis was applied to normalized (reads per kilobases of transcript per 1 million mapped reads) and log-transformed count data. (B) Hierarchical clustering was performed using DESeq2 based on Poisson distance. The colored scale bar represents sample distances (blue signifies a high correlation). (C) A Cook's distance boxplot was constructed to visualize the data outlier detection and removal by DESeq2. WT, wild-type epimastigotes; OE, overexpression of TcUBP1.

SUPPLEMENTAL FILES

Supplemental File 1. List of 1,164 affected genes after UBP1 overexpression with >2-fold change and FDR < 0.05. Of these, 793 genes were up-regulated and 371 were down-regulated.

Supplemental File 2. List of exclusively expressed genes in WT or UBP1-OE samples.

Supplemental File 3. Gene ontology defined by TriTrypDB functional analysis tool.

Supplemental File 4. Complete list of gene ontology gene clusters defined by DAVID server.

Supplemental File 5. GeneID list of 1737 genes used for PC analysis depicted in Fig. 7.

Supplemental File 6. Complete list of different types of *T. cruzi* RBPs used for bioinformatic RPI predictions.

Supplemental File 7. List of GeneID, protein names and fold change of RBPs coding transcripts affected after TcUBP1 overexpression, with |log2 fold change| > 0.58.
